# COVID-19-Associated Rhino-Orbito-Cerebral Mucormycosis: A Single Tertiary Care Center Experience of Imaging Findings With a Special Focus on Intracranial Manifestations and Pathways of Intracranial Spread

**DOI:** 10.7759/cureus.57441

**Published:** 2024-04-02

**Authors:** Megha G Nair, Shilpa Sankhe, Gayatri Autkar

**Affiliations:** 1 Department of Radiodiagnosis, Seth Gordhandas Sunderdas Medical College (GSMC) King Edward Memorial (KEM) Hospital, Mumbai, IND

**Keywords:** rhino-orbito-cerebral, black fungus, imaging findings, rhinocerebral mucormycosis, covid-19 retro

## Abstract

Background and objective

The COVID-19 pandemic and mucormycosis epidemic in India made research on the radiological findings of COVID-19-associated mucormycosis imperative. This study aims to describe the imaging findings in COVID-19-associated mucormycosis, with a special focus on the intracranial manifestations.

Methodology

Magnetic resonance imaging (MRI) scans of all patients with laboratory-proven mucormycosis and post-COVID-19 status, for two months, at an Indian Tertiary Care Referral Centre, were retrospectively reviewed, and descriptive statistical analysis was carried out.

Results

A total of 58 patients (47 men, 81%, and 11 women, 19%) were evaluated. Deranged blood glucose levels were observed in 47 (81%) cases. The intracranial invasion was detected in 31 (53.4%) patients. The most common finding in cases with intracranial invasion was pachymeningeal enhancement (28/31, 90.3%). This was followed by infarcts (17/31, 55%), cavernous sinus thrombosis (11/58, 18.9%), fungal abscesses (11/31, 35.4%), and intracranial hemorrhage (5/31, 16.1% cases). The perineural spread was observed in 21.6% (11/51) cases. Orbital findings included extraconal fat and muscle involvement, intraconal involvement, orbital apicitis, optic neuritis, panophthalmitis, and orbital abscess formation in decreasing order of frequency. Cohen’s kappa coefficient of interrater reliability for optic nerve involvement and cavernous sinus thrombosis was 0.7. Cohen’s coefficient for all other findings was 0.8-0.9.

Conclusions

COVID-19-associated rhino-orbito-cerebral mucormycosis has a plethora of orbital and intracranial manifestations. MRI, with its superior soft-tissue resolution and high interrater reliability, as elucidated in this study, is the imaging modality of choice for expediting the initial diagnosis, accurately mapping out disease extent, and promptly identifying and scrupulously managing its complications.

## Introduction

The COVID-19 pandemic ravaged most nations across the world [1]. Following the second wave, however, there was a cascade of mucormycosis cases in India [2]. This previously uncommon fungal infection explosively increased in numbers. Filamentous fungi that belong to the subphylum Mucoromycotina and order Mucorales cause an invasive disease, which may involve multiple systems, namely, the paranasal sinuses, the orbits, the central nervous system, musculoskeletal system, lungs, gastrointestinal tract, and even the liver [3-5]. This is denoted by the term *mucormycosis*. It is often called rhino-orbito-cerebral mucormycosis based on the most frequently invaded organs [3]. Mucormycosis is already the most rapidly fatal fungal infection in human beings [6]. This study was an attempt to explore and objectively characterize the imaging manifestations in laboratory-confirmed cases of COVID-­19-associated rhino-orbito-cerebral mucormycosis [7]. This study aimed to describe the imaging findings in COVID-19-associated rhino-orbito-cerebral mucormycosis, with a special focus on the intracranial manifestations, and pathways of spread and determine the interrater reliability of the various parameters used in the evaluation of rhino-orbito-cerebral mucormycosis.

An earlier version of this study was posted to preprint server researchsquare.com on February 25, 2022.

## Materials and methods

This study included 58 laboratory-confirmed cases of COVID-19-associated rhino-orbito-cerebral mucormycosis, fulfilling the inclusion criteria, who underwent contrast-enhanced magnetic resonance imaging (MRI) at our institute between March 15, 2021, and November 15, 2021. Approval from the Institutional Review Board and Institutional Ethics Committee was obtained for this retrospective study. We reviewed patients' imaging studies and departmental records. A consent waiver was granted by the institutional ethics committee because of its retrospective nature.

Imaging technique

The standard protocol followed at our institute for suspected or diagnosed cases of mucormycosis was adhered to. MRI was performed on a Siemens Magnetom Aera 1.5-Tesla magnet with a 48-channel head coil equipped with array spatial sensitivity encoding and parallel acquisition. Our standard orbit, paranasal sinus, and brain MRI protocol include the following sequences: whole-brain axial turbo spin-echo T2-weighted, fluid-attenuated inversion recovery (FLAIR) coronal, axial susceptibility-weighted (SWI), T1-weighted non-fat-saturated sagittal, diffusion-weighted imaging (DWI) B1000, and the generation of trace and Apparent Diffusion Coefficient (ADC) maps. Axial and coronal brain T1 fat-saturated contrast-enhanced sequences were also acquired. High-resolution orbit sequences include fast spin-echo axial T2, unenhanced axial T1, contrast-enhanced axial T1, and coronal images also covering the paranasal sinuses. A post-contrast Magnetization Prepared Rapid Acquisition Gradient Echo (MPRAGE) sequence was also acquired. Time of flight (TOF) brain and neck angiography was performed in patients showing infarcts or abnormal internal carotid artery (ICA) flow voids. We used a standard dose of 0.1 mmol/kg of gadoterate meglumine, an intravenous contrast material (Dotarem, Guerbet, France). In patients with intracranial hemorrhage, the protocol was extended to include MRI venography.

Eligibility criteria

Patients with documented reverse transcriptase-polymerase chain reaction (RT-PCR)-positive status within the past two months and laboratory-proven mucormycosis either on potassium hydroxide (KOH) mount, fungal culture, or histopathology were included. Patients who had undergone treatment including debridement or medical management before imaging and scans of suboptimal quality due to artifacts were excluded from the study.

Image analysis

The studies were independently reviewed on a Siemens Syngo standalone viewer, version VD11 (Erlangen, Germany), by two qualified radiologists with three and five years of experience, respectively. Disagreements were resolved independently by a third senior radiologist with 15 years of experience. CT data of these patients were retrospectively analyzed only to evaluate bone involvement or to look for the presence of hemorrhage. We followed a structured reporting format in all cases of COVID-19-associated mucormycosis.

The assessment primarily utilized contrast-enhanced MRI scans to evaluate various structures, including the maxillary, ethmoid, sphenoid, and frontal sinuses, turbinates, lamina papyracea, cribriform plate, sphenoid bone, and pterygoid plates for mucosal changes, altered signal intensity, and post-contrast enhancement. CT images were also reviewed for bone erosions. Additionally, soft tissues like the pterygomaxillary fissure, pterygopalatine fossa, retro-antral fat, masticator space muscles, subcutaneous tissues, extraocular muscles, extra-conal fat, intraconal fat, and orbital apex were assessed for signal intensity variations and the presence of enhancing soft tissue. The pathways of spread were inferred from the contiguous sites of involvement. Non-contrast and contrast-enhanced sequences of brain MRI were evaluated in detail, for all suspected complications. Both source images and maximum intensity projection (MIP) images of TOF angiography were evaluated.

Statistical analysis

Statistical analysis was performed using IBM SPSS Statistics for Windows, Version 27.0 (IBM Corp., Armonk, NY). Numerical data have been analyzed and expressed as mean (standard deviation) and range. Ratios, proportions, and percentages were derived for categorical and nominal data. Interrater agreement was also assessed for the two primary reviewers. Cohen’s kappa coefficient was assessed. The interpretation of Cohen’s kappa coefficient was as follows: values ≤ 0 indicated no agreement, 0.01-0.20 indicated no to slight agreement, 0.21-0.40 indicated fair agreement, 0.41-0.60 indicated moderate agreement, 0.61-0.80 indicated substantial agreement, and 0.81-1.00 indicated near-perfect agreement [8].

## Results

Demographics

A total of 58 cases fulfilling the inclusion criteria were selected for the study. The mean age of the study population was 49.2 (range 18-77) years. The standard deviation was 12.7 years. The male-to-female ratio of patients was 47:11, that is, 81% of patients were men and 19% were women. All the patients included in the study had complaints of either facial pain or facial swelling along with previous COVID-19-positive status. 

Clinical history and laboratory findings

Among the patients included in the study, 37 (64%) were known diabetics and the rest had no documented evidence of deranged blood sugars or diabetes before the COVID-19 episode. One patient was a case of renal transplantation on immunosuppression and another had acute promyelocytic leukemia, apart from their previous COVID-19-positive status. Out of these 58 cases, 47 (81%) had documented deranged blood glucose levels during the COVID-19 episode or thereafter.

Imaging findings

Ethmoid sinus (57/58, 98.3%) was found to be the most frequently involved sinus. Here, bilateral involvement (49/58, 84.5%) was more common than unilateral involvement. This was followed by maxillary sinus involvement. Among the turbinates, the middle turbinate was found to be the most frequently involved. Pterygomaxillary fissure (37/58, 64.0%) and pterygopalatine fossa (33/58, 56.9%) were equally involved on the right and the left. Unilateral involvement of the pterygomaxillary fissure is much more common than bilateral involvement. The most commonly involved muscle in the masticator space was the medial pterygoid muscle. The retro-antral fat pad was also nearly equally affected on both sides, namely, 21/58 on the right side and 20/58 on the left side. Of the 58 cases, 33 revealed direct spread into the retroantral fat pad and pterygopalatine fossa without any bony erosion. 

Subcutaneous extension into the premaxillary fat or the preseptal region of the orbit was seen in 40 (70%) cases. Parotid gland involvement was seen in a single case. The perineural spread was seen in 11 cases (19%), and the left mandibular nerve (8/58, 13.8% cases) was found to be the most common nerve demonstrating the perineural spread. The orbital invasion was found to be predominant in the extraconal space on the medial aspect (36/58, 62.1%) followed by the inferior aspect (30/58, 51.7%). Unilateral involvement was found to be far more common than bilateral involvement. Correspondingly, the most commonly involved intraocular muscles were also found to be the medial rectus (38/58, 65.5%), and the inferior rectus muscles (23/58, 39.7%) closely followed by the superior rectus (21/58, 36%). The involvement of orbit and related structures are elaborated in Table 1. An orbital abscess was also seen in one case, which was later on drained surgically.

**Table 1:** Involvement of orbit and related structures.

Structures involved	Right	Left	Both
Extraconal fat	17	25	6
Intraconal fat	12	16	7
Infraorbital nerve	5	12	13
Apex	10	17	4
Optic nerve	10	16	3
Lamina papyracea	14	13	4
Cribriform plate	22		
Panophthalmitis	2	1	0
Orbital abscess	0	1	0

Intracranial findings

Of the 58 patients, 31 (53.4%) were found to have intracranial invasion. Pachymeningeal enhancement (28/31, 90.3%) was found to be the most common observation, indicating intracranial invasion. Infarcts (17/31, 55%) were the second most common finding, indicating intracranial invasion. Among the cases with infarcts, four were watershed territory infarcts occurring in cases with complete ICA thrombosis. Cavernous sinus thrombosis (11/58, 18.90%) and internal carotid artery involvement (11/58, 18.90%) were also seen. Among the cases with internal carotid artery involvement, 8/11 (72.7%) had complete thrombosis of the internal carotid artery right from the origin. Of the 11 cases, 3 (27.2%) showed internal carotid artery wall thickening, enhancement, and narrowing.

Fungal intraparenchymal abscesses or changes of cerebritis were more common than fungal extra-axial abscesses. Intraventricular exudates were also seen in two cases in the dependant portion with restricted diffusion on DWI, i.e., the occipital horn of both lateral ventricles. Extra-axial hemorrhage in the form of subdural and subarachnoid hemorrhage and intraparenchymal hemorrhage was seen in two and four cases, respectively. Hence, a total of 6/31 (19.5%) cases showed hemorrhagic changes. The frequency distribution of intracranial findings and other manifestations in the study group is demonstrated in Table 2. The imaging appearances of various complications of rhino-orbito-cerebral mucormycosis are listed in Table 3. These criteria were used to determine Cohen’s kappa coefficient for the interrater agreement for various parameters. The Cohen’s kappa coefficient for the optic nerve involvement and cavernous sinus thrombosis was 0.7, suggestive of substantial interrater agreement; all other findings had a Cohen’s coefficient value in the range of 0.8-0.9, suggestive of near-perfect agreement.

**Table 2:** Intracranial and neurovascular manifestations.

Criteria	Number of patients, *n*	%
Total number of patients	58	100
Intracranial invasion	31	53
Pachymeningeal enhancement	28	48
Cavernous sinus thrombosis	11	18.90
Internal carotid artery involvement	11	19
Internal carotid artery thrombosis	8	13.70
Internal carotid artery wall thickening, enhancement, and narrowing	3	5.10
Infarcts	17	29
Watershed territory infarcts	4	6.90
Vasculitic/embolic infarcts	12	20.6
Extra-axial hemorrhage	2	3.40
Intraparenchymal hemorrhage	4	6.90
Fungal intraparenchymal abscess	7	12.00
Fungal extra-axial abscess	5	8.60
Mycotic aneurysm	2	3.40
Intraventricular exudate	2	3.40

**Table 3:** Imaging appearance of various complications of rhino-orbito-cerebral mucormycosis. CSF, cerebrospinal fluid; FLAIR, fluid-attenuated inversion recovery

Complication	Imaging appearance
Cavernous sinus thrombosis	Presence of convex outer walls. Signal characteristics vary depending on the age of the thrombus but will be abnormal. Post-contrast images were assessed for any filling defect within the sinus and the presence of abnormal dural enhancement along its lateral wall.
Angioinvasive disease involving the internal carotid artery	T2 hyperintensity of the wall with wall thickening, enhancement, narrowing, and luminal irregularity involving the internal carotid artery.
Mycotic aneurysm	Eccentric saccular or fusiform aneurysm with changes of vasculitis or enhancing soft tissue surrounding it.
Panophthalmitis	Abnormal shape of the eyeball with diffusion restriction within the posterior coats as well as heterogenous contents in the eyeball.
Optic nerve involvement	Swelling of the optic nerve with obliteration of the CSF sleeve and T2/FLAIR hyperintense signal with post-contrast enhancement.
Perineural spread	Thickening of the nerve, widening of the adjacent neural foramen, and enhancement along the nerve.

## Discussion

The focus of this study was on the intracranial and orbital manifestations of COVID-19 associated with rhino-orbito-cerebral mucormycosis. The role of MRI in the evaluation of mucormycosis has been well described previously, and a few recent studies have also detailed the intracranial manifestations of mucormycosis on a backdrop of COVID-19 [9,10]. Early signs of mucormycosis include the presence of non-enhancing turbinates, described as the black turbinate sign [11,12]. Similar findings were also seen in our study. Our findings included a significantly heterogeneous appearance within the involved sinuses, including T1 isointensity or hyperintensity, as well as T1/T2 hypointensity or isointensity.

Patients with diabetes have already been known to be more prone to mucormycosis [4]. The European registry of patients with mucormycosis has also revealed a strong association between mucormycosis and diabetes as well as corticosteroid use [13,14]. There is also evidence of an association between diabetes and COVID-19 infection, including the triggering of diabetic ketoacidosis [15,16]. COVID-19 itself may also predispose to invasive fungal infections, especially of the sinuses [17,18]. By demonstrating the association of these conditions, this study gives objective clues regarding the sudden surge in mucormycosis cases [19].

Before the COVID-19 pandemic, mucormycosis had a higher incidence in the Indian subcontinent than in any other region of the world, likely related to the higher burden of diabetes in India [20]. Susceptibility to rhino-orbito-cerebral mucormycosis in patients with COVID-19 is a consequence of decreased phagocytic activity and increased accessible iron. Protons in diabetic ketoacidosis stimulate a transferrin-mediated displacement of iron. Fungal heme oxygenase promotes iron absorption for fungal growth. This iron dependence also explains the angioinvasive propensity of fungal pathogens [21]. Our study also included a case of post-COVID-19 rhino-orbito-cerebral mucormycosis in a patient with a history of solid organ transplantation as well as one with a hemo-lymphatic malignancy [22]. Similar phenomena have been documented previously [14,23,24]. However, this has rarely been reported in a post-COVID-19 context [22].

Our study predominantly utilized MRI for the evaluation of rhino-orbito-cerebral mucormycosis. MRI not only possesses excellent soft-tissue resolution but is also free from ionizing radiation. MRI is more likely to be safe in the evaluation of these patients because patients with mucormycosis often need the administration of nephrotoxic drugs [12]. CT was reviewed to retrospectively evaluate the bony structures and help distinguish between susceptibility due to hemorrhage versus susceptibility due to fungal elements. Our study also revealed a large number of cases in which there was spread of the disease without underlying bone erosion. This is because the intrinsic pathway of spread is along blood vessels or nerves and does not necessarily require bone erosion [20]. Skull base osteomyelitis is also well delineated on MRI.

There are several unanswered questions regarding the pathways of the spread of mucormycosis. Mucormycosis initially involves the nasal mucosa. This is followed by spread to the paranasal sinuses (typically the ethmoid and maxillary sinuses), then the orbit, and ultimately the intracranial fossa [25]. Ethmoid sinuses were found to be most frequently involved in our study. This is in keeping with the findings described in a recent study [26]. The frequency of involvement of structures may also help in determining the temporal evolution of the disease process. The direct pathways of invasion of various structures observed in our study also help to advance the understanding of the pathogenesis of mucormycosis.

According to our study, the dominant pathway for the spread of mucormycosis is across the ethmoidal air cells and the lamina papyracea into the orbit and then into the medial and inferior aspect of the extraconal fat. Subsequently, the medial and inferior rectus muscles are involved. However, the inferior aspect of the extraconal fat of the orbit may also be invaded across the orbital floor. The superior rectus muscle is typically involved via the spread into the superior extraconal space. This is also mostly through the lamina papyracea and occasionally via the frontal bone from the frontal sinus through the superior wall of the orbit.

Invasion of orbital apex following intraconal invasion may result in orbital apex syndrome. Optic neuritis may ensue. Invasion of the superior orbital fissure, which contains cranial nerves III, IV, and VI, and branches of V1 and V2, may result in ophthalmoplegia, diplopia, paraesthesia, and loss of sensations in the corresponding territories of the cornea and face. All cases of cavernous sinus thrombosis in our study had associated orbital apex involvement. This is in keeping with common pathways of spread into the cavernous sinus [25,27]. The pathways of intracranial spread most frequently included the orbital apex, with cavernous sinus involvement spreading into the adjacent middle cranial fossa, medial to the anterior temporal lobe with pachymeningeal enhancement, and the presence of extra-axial and intraparenchymal abscesses in this region [28].

Another pathway of spread is across the cribriform plate and the basifrontal region (anterior cranial fossa extension), causing subdural abscesses and adjacent vasculitic infarcts as well as intraparenchymal abscesses [29]. All cases of intraparenchymal and extra-axial hemorrhages in our study were located in the basifrontal region. Intracranial hemorrhage was probably due to mycotic aneurysms and angioinvasive disease. We observed mycotic aneurysms in the cavernous ICA, as well as in the posterior cerebral artery.

We also observed contiguous involvement of the infraorbital nerve in 30 (51.7%) patients. This was found to cause the widening of the infraorbital foramen. The disease spread was then found to be along the inferior orbital fissure progressing to the pterygopalatine fossa. A few cases also showed extension across the foramen rotundum into the middle cranial fossa. Intracranial extension into the posterior cranial fossa was also demonstrated with vasculitic infarcts in the left superior cerebellar artery territory. There was also a case of a right middle cerebellar peduncle abscess. This represented extension along the cisternal segment of the right trigeminal nerve. Only a few reports have documented this phenomenon [6]. It indicates a pathway of perineural spread of the disease directly into the posterior cranial fossa [30].

Diffusion restriction is often seen and helps in evaluating the extent of involvement of various structures including the optic nerve, orbital apex, and the intracranial spread [12]. Fungal abscesses may present with only peripherally restricted diffusion or heterogeneous diffusion restriction within the abscesses. Infarcts included vasculitic or embolic infarcts as well as watershed territory infarcts in cases of complete ICA thrombosis.

In previous studies on non-COVID-19-associated mucormycosis, the most commonly documented findings in the intracranial extension of mucormycosis, apart from meningeal enhancement, have been intracranial infected soft tissue/abscesses in 50% of patients with intracranial extension and infarcts only in 20% of cases. Previous studies have demonstrated 31% involvement of intracranial structures out of which 38% showed cerebritis or abscesses and 30% cases showed infarcts [10,11]. In comparison, our study, where 31 cases showed intracranial involvement, demonstrated infarcts in 55% of cases and internal carotid artery thrombosis in 13.8% of cases. This suggests a possibility of a higher occurrence of arterial thrombotic events, including carotid artery involvement and infarcts in COVID-19-associated mucormycosis.

We have only considered acute infarcts with diffusion restriction on DWI in this study. Given the association of COVID-19 with various thrombotic phenomena and the findings of our study, the possibility of a higher frequency of thrombotic complications in COVID-19-associated mucormycosis vis-à-vis the proportion of these complications in patients with mucormycosis alone merits consideration. All cases of infratemporal fossa involvement in our study also had ipsilateral pterygomaxillary fissure involvement. This corroborates the theory of spread into the infratemporal fossa via the pterygomaxillary fissure [30].

Panophthalmitis and optic neuritis are devastating complications of rhino-orbito-cerebral mucormycosis and may result in permanent blindness. Early detection and prompt management of these complications may help to preserve vision. However, in some cases with panophthalmitis, orbital exenteration may be needed. Here too, MRI plays a crucial role in the decision-making process. The limitations of our study include the absence of a control group.

## Conclusions

It is of paramount importance for clinicians as well as radiologists to consider the possibility of rhino-orbito-cerebral mucormycosis and evaluate in-depth for its myriad of possible complications. COVID-19-associated rhino-orbito-cerebral mucormycosis has a plethora of intracranial manifestations. MRI, with its superior soft-tissue resolution and high interrater reliability of findings, as elucidated in our study, can not only expedite the diagnosis of the initial stages of mucormycosis and help in the early diagnosis of its complications but can also guide the clinician by effectively mapping out the exact disease extent. Varied pathways of intracranial spread have been noted, including direct invasion, vascular spread, perineural spread across different nerves, and neural foramina. Given the findings of our study and the association of COVID-19 with various thrombotic phenomena, we propose the hypothesis that post-COVID-19 mucormycosis may be associated with a larger number of acute infarcts in patients with intracranial involvement as compared to the intracranial complications occurring in patients with mucormycosis alone. This requires validation with larger studies and may have important therapeutic implications.
